# The optimal starting time of postoperative intraperitoneal mitomycin-C therapy with preserved intestinal wound healing

**DOI:** 10.1186/1471-2407-5-31

**Published:** 2005-03-31

**Authors:** Ali Uzunkoy, Cengiz Bolukbas, Mehmet Horoz, Fusun F Bolukbas, Abdurrahim Kocyigit

**Affiliations:** 1 Department of Surgery, Harran University, Sanliurfa, Turkey; 2 Department of Internal Medicine, Harran University, Sanliurfa, Turkey; 3 Department of Biochemistry, Harran University, Sanliurfa, Turkey

## Abstract

**Background:**

There is controversy about the effect of the timing of intraperitoneal administration of chemotherapeutic agents on the healing of intestinal anastomosis. We have investigated the effect on intestinal wound healing of mitomycin-C administered at different times post-operatively.

**Methods:**

Eighty-four Wistar-Albino female rats underwent ileal resection and end-to-end anastomosis. The rats were randomly selected for intraperitoneal administration of mitomycin-C or saline as follows: mitomycin-C group (n = 65), 2 mg/kg mitomycin-C; control group (n = 13), 10 ml saline. The former was sub-divided into 5 equal groups (A 1–5) and mitomycin-C was administered postoperatively as follows: day 0 (A1), day 3 (A2), day 5 (A3), day 7 (A4) and day 10 (A5). All the rats were sacrificed on the 14th postoperative day and anastomotic bursting pressures and tissue hydroxyproline levels were determined.

**Results:**

Five of the animals died postoperatively: 2 (15.4%) in group A1, 2 (15.4%) in group A2 and 1(7.7%) in group A3. Non-lethal anastomotic leakage was observed in a further five animals: 1 in group A1, 2 in group A2, 1 in group A5 and 1 in the control group. Groups A1 and A2 had significantly lower anastomotic bursting pressures than the other groups (P was <0.05 for each comparison). The anastomotic bursting pressures of group A3, A4 and A5 were comparable with those of the controls (P was >0.05 for each comparison). Tissue hydroxyproline levels in group A1 and A2 were significantly lower than in the controls (P values were <0.05 for each comparison) or the other mitomycin-C sub-groups (P was <0.05 for each comparison).

**Conclusions:**

Intraperitoneal chemotherapy impairs intestinal wound healing when applied before the 5th postoperative day. Additional therapeutic approaches are needed to prevent this potentially lethal side effect of early intraperitoneal mitomycin-C administration.

## Background

Intraperitoneal (IP) administration of antineoplastic agents has been studied in many investigative trials and has been used in clinical practice for more than 40 years [1]. Serosal invasion occurring in spite of curative resection is an unfavourable prognostic factor in gastrointestinal malignancies [2]. IP chemotherapy is now viewed as an interesting approach to treating tumors with peritoneal, local lymphatic or hepatic dissemination [3] such as gastric or colon cancer. The main goal of IP administration is to increase the exposure of cancer cells in the peritoneal cavity to the drugs with minimal potential toxic effects on the internal organs [1,4,5]. Various chemotherapeutic agents such as mitomycin-C (MTC), cisplatin and 5-fluorouracil, administered intraperitoneally during the early postoperative period, have been shown to be safe and to improve prognosis in patients with gastrointestinal cancers [6,7].

The timing as well as the route of administration of the chemotherapeutic agent plays a major role in determining the response. Since drug resistance is often related to the number of neoplastic cells present when the chemotherapeutic treatment is initiated, early administration of the agent in conjunction with surgery may give better results [8]. However, the effect of chemotherapeutic agents, delivered intravenously or intraperitoneally, on the healing of intestinal anastomosis is still controversial [9-16]. Weiber [14] investigated the influence of postoperative 5-fluorouracil on intestinal anastomotic wound healing in relation to timing of administration, but no other chemotherapeutic agents have been studied in this way. Our aim in the present study was to investigate the effects of IP MTC, administered at various times postoperatively, on intestinal anastomotic wound healing. MTC has been extensively used for treating gastric cancers.

## Methods

Eighty-four Wistar-Albino female rats weighing 275 ± 25 g were acclimatized for 10 days before the study. All the animals were in good health and were kept under the same physical and environmental conditions. They were fed with standard pellet diet and tap water and kept at 23 ± 2°C, with a 12 h light/dark cycle, throughout the study period. They received human care and the study protocol complied with the institutions' guidelines. All the rats underwent abdominal surgery under ether anesthesia. Standardized laparotomy was performed through a 3-cm midline incision. A 2-cm ileal segment was resected up to 10 cm proximal to the ileocecal valve. An end-to-end anastomosis was made using interrupted 5/0 polypropylene sutures as a continuous single layer. The fascia was closed using interrupted 3/0 polypropylene sutures. Subsequently, the rats were randomly assigned to two groups: group A, MTC group(n = 65); control (saline) group (n = 13). The MTC group was sub-divided into 5 equal (n = 13) groups, A1-A5, and IP mitomycin-C was administered postoperatively as follows: day 0 (A1), day 3 (A2), day 5 (A3), day 7 (A4) and day 10 (A5). Each MTC dosage was 2 mg/kg with 10 ml saline. All the rats were sacrificed with an overdose of ether on the 14th postoperative day. Postmortem exploration of the abdomen to detect anastomotic complications was performed by an observer blinded to the treatment arm of the study. The entire anastomotic segment of ileum with 2-cm margins on either side was removed for study.

### Anastomotic bursting pressure measurement

Anastomotic bursting pressure was measured in all rats. A catheter was inserted intraluminally into the resected segment of the ileum and secured using pursestring sutures. A mercury manometer connected to an inflating apparatus was attached to the other side of the catheter and the open end of the ileum was ligated. The resected segment of the intestine was then totally submerged in a water pot and gently distended with air. The bursting pressure was recorded as mmHg at the time of appearance of the first gas bubble.

### Hydroxyproline content determination

Tissue hydroxyproline content was determined in all rats by the Reddy method [17] and the results were recorded as μg hydroxyproline per mg dry tissue weight.

### Statistical analysis

Non-parametric continuous variables were compared by the Kruskal-Wallis one-way analysis of variance with post-hoc analysis using a Mann-Whitney U test. Parametric variables were compared using one-way analysis of variance with post-hoc analysis using the Tukey test. Data are presented as medians and ranges for nonparametric variables and means ± SD for parametric variables. Differences were regarded as significant at p < 0.05 for parametric and 0.05/6 for nonparametric analyses.

## Results

Postoperative mortality was observed in five animals: 2 (15.4%) in group A1, 2 (15.4%) in group A2 and 1 (7.7%) in group A3. The deaths in group A1 occurred on the 1st postoperative day; the other deaths occurred before administration of MTC at the 2nd postoperative day. Macroscopic examination during post-mortem laparotomy showed anastomotic leakage communicating with abdominal abscesses in all deaths. Because the deaths in group A2 an A3 occurred before MTC administration and those in group A1 occurred earlier, the anastomotic leakage was attributed to operative complication and the dead rats were excluded from the study. No mortality occurred in any of the remaining animals before the completion of the study. During post-mortem abdominal explorations at the end of the study, non-lethal anastomotic leakage was observed in 5 animals: 1 in group A1, 2 in group A2, 1 in group A5 and 1 in the control group.

### Anastomotic bursting pressure

**Figure 1:**
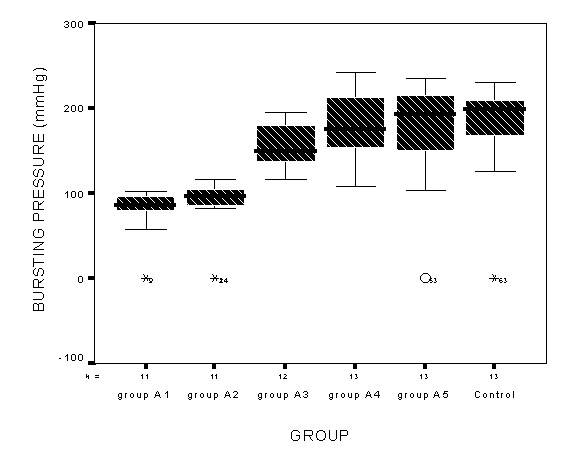
Anastomotic bursting pressures in mitomycin-C sub-groups and controls. Group A1 and A2 which have received mitomycin-C (MTC) in preoperative (day 0) and 3rd day of postoperative period, respectively, had significantly lower value than the other groups (both, p < 0.001).

Anastomotic bursting pressures (mmHg) were 86 (0–102), 96 (0–116), 149.5 (116–196), 175 (108–241), 193 (0–243) and 198 (0–231) in groups A1, A2, A3, A4, A5 and control, respectively (Fig. 1). The values in groups A1 and A2 were significantly lower than in groups A3, A4, A5 and control (P values were <0.05 for all these comparisons). There was no statistically significant difference between groups A1 and A2 (p > 0.05). The anastomotic bursting pressures in groups A3, A4 and A5 were not significantly different from those in the control group (P values were >0.05/6 for these comparisons).

### Tissue hydroxyproline content

**Figure 2:**
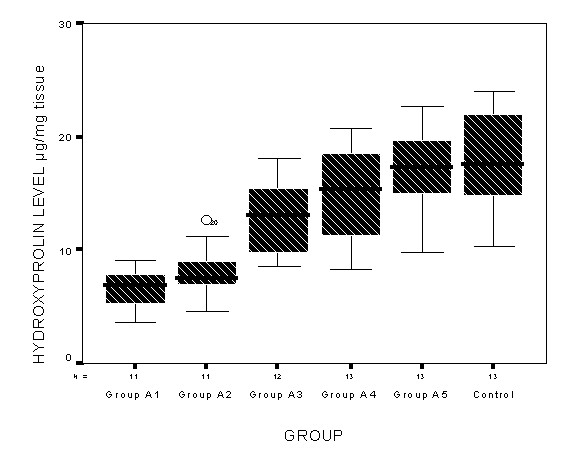
Tissue hydroxyproline levels in mitomycin-C sub-groups and controls. Group A1 and A2 which have received mitomycin-C in preoperative (day 0) and 3rd day of postoperative period, respectively, had significantly lower value than the other groups (both, p < 0.001).

The mean tissue hydroxyproline contents (μg/mg) of groups A1, A2, A3, A4, A5 and control were 6.54 ± 1.9, 8.14 ± 2.3, 14.77 ± 3.4, 14.80 ± 4.3, 17.01 ± 3.9 and 18.28 ± 4.3, respectively (Fig. 2). The values for groups A3, A4 and A5 were comparable with the controls (P values were >0.05 for these comparisons). The tissue hydroxyproline levels of groups A1 and A2 were significantly lower than the controls or the other MTC sub-groups (P values were <0.05 for these comparisons).

## Discussion

Recurrence and IP dissemination of gastrointestinal cancers is common despite curative resection, particularly when the serosa has been infiltrated [2]. It has been suggested that IP administration of anticancer agents before closure of the laparotomy, and during the first few postoperative days, may prevent the dissemination of cancer cells [18-20]. A potential criticism of delayed postoperative IP administration is inhomogeneous drug distribution caused by early postoperative adhesions [21]. Entrapment of the tumor cells by fibrin in the dissected areas decreases their exposure to the chemotherapeutic agents [22,23]. Thus, the use of IP chemotherapy at the time of surgery provides better drug distribution without the risk of interference from postoperative adhesions [21]. However, although the administration of chemotherapeutic agents in the immediate postoperative period may decrease the local cancer recurrence rate, this must be weighed against the possible impairment of wound healing [12,24,25].

In the present study, the animals that received IP MTC perioperatively and on the 3rd potoperative day had significantly lower mean tissue hydroxyproline contents and anastomotic bursting pressures than the controls. No difference was observed between the controls and the other MTC sub-groups. Collagen synthesis, which may be reflected by tissue hydroxyproline content, is an essential feature of anastomotic healing in the intestine [17,26]. We therefore measured the tissue hydroxyproline content to determine whether MTC is detrimental to anastomotic wound healing. The data showed that IP MTC administered perioperatively or on the 3rd postoperative day was detrimental to the healing of intestinal anastomosis, but MTC administered on or after the 5th postoperative day had no significant effect on the process. The effects of IP administration of various chemotherapeutic agents on intestinal anastomotic wound healing have been investigated in many studies and the results have been conflicting [12-14,16,24,27,28]; some have shown detrimental effects [12,28]. However, the effect on wound healing of peri- and post-operative intraperitoneal MTC administration has not been described previously.

Tissue injury initiates a cycle of inflammation, cellular migration and replication, and connective tissue deposition and remodeling, which restores tissue integrity. Successful wound healing depends on the formation of a strong and stable scar. Wound strength is determined by the amount and quality of newly-synthesized and deposited collagen, and by the degradation of preformed collagen [29]. Collagenase activity, which is important in determining anastomotic integrity and suture-holding capacity during the first few days of healing, increases significantly after 3 days of colonic anastomosis; at this time the suture holding capacity of the anastomosis decreases by up to 80% [30-33]. MTC arrests the proliferation of fibroblasts, which are responsible for several crucial aspects of the wound-healing process outlined above [34]. Thus, IP administeration of MTC within three days of the operation may be detrimental to intestinal anastomotic wound healing, as shown in the present study.

Ineffective colonic wound healing can have devastating consequences e.g. abscesses, fistula formation, or death secondary to overwhelming sepsis. Therefore, although early IP administration of MTC following cytoreductive surgery is effective in preventing the dissemination of cancer cells, the side effects discussed here have considerable importance and should not be overlooked.

In conclusion, certain interventions are needed to avoid the potentially lethal side effects of chemotherapy on intestinal wound healing in subjects receiving early IP administration. Additional therapeutic approaches such as anti-inflammatory or fibrinolytic agents should be introduced to reduce adhesion formation, and thus preclude inhomogeneous drug distribution, in patients for whom IP administration has been postponed beyond the early postoperative period to avoid the undesirable side effects.

## Abbreviations

IP, intraperitoneal; MTC, mitomycin-C.

## Competing interests

The author(s) declare that they have no competing interests.

## Authors' contributions

AU conceived the study and participated in its design and coordination. CB, MH and FFB conceived the study, participated in the sequence alignment and drafted the manuscript. AK collected the samples and carried out the laboratory analysis.

## Pre-publication history

The pre-publication history for this paper can be accessed here:
